# Supplementary material for: The causal relationship between the human gut microbiota and pyogenic arthritis: a Mendelian randomization study
Source: Front Cell Infect Microbiol. 2024 Nov 26;14:1452480. doi: 10.3389/fcimb.2024.1452480 (PMC11629706; doi:10.3389/fcimb.2024.1452480)
Supplement: Supplementary file 1 [file DataSheet1.zip › mendelian test/ebi-a-GCST90027844.csv_forest.pdf]

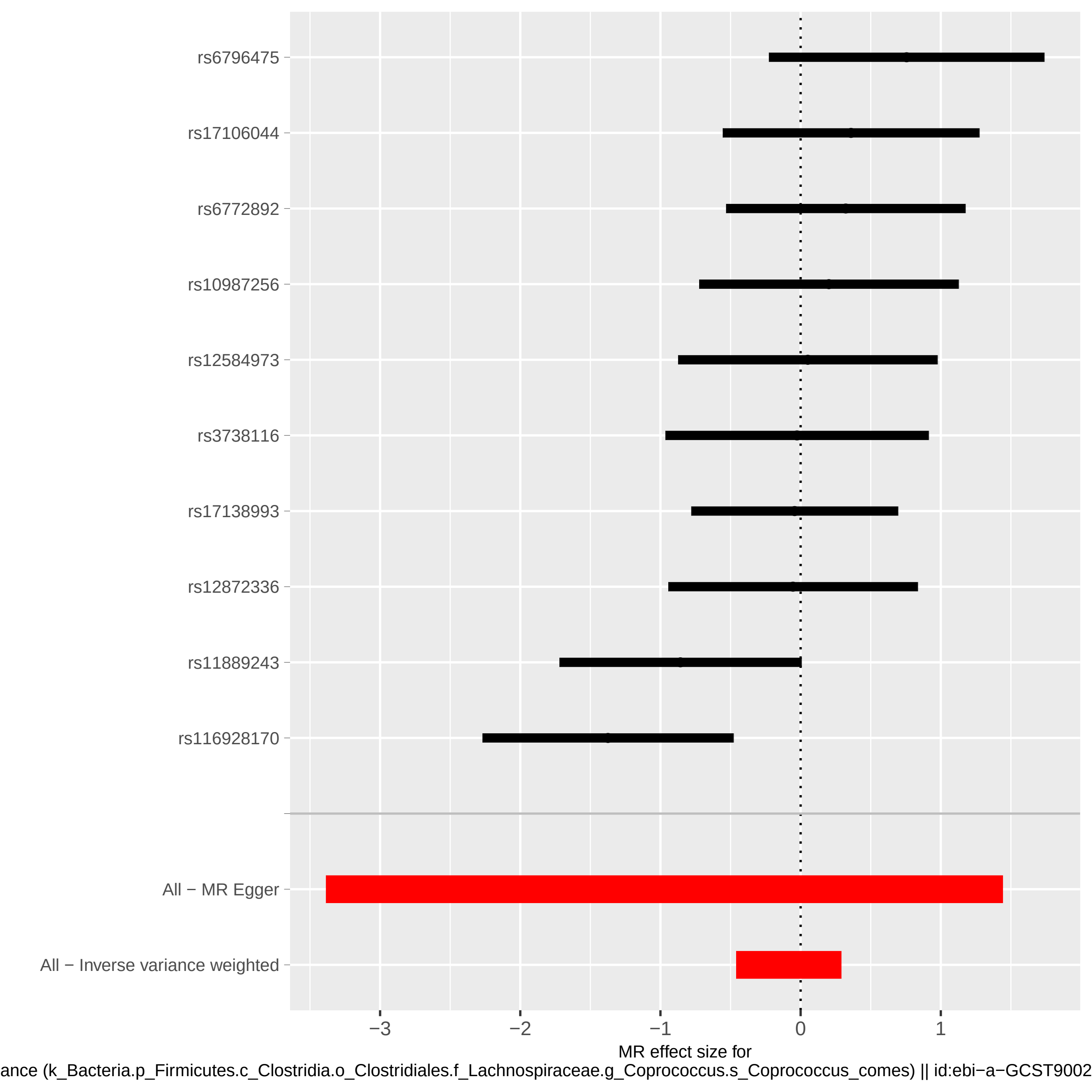
rs6796475
rs17106044
rs6772892
rs10987256
rs12584973
rs3738116
rs17138993
rs12872336
rs11889243
rs116928170
All − MR Egger
All − Inverse variance weighted
−3
−2
−1
0
1
MR effect size for
ance (k_Bacteria.p_Firmicutes.c_Clostridia.o_Clostridiales.f_Lachnospiraceae.g_Coprococcus.s_Coprococcus_comes) || id:ebi−a−GCST9002
